# Clinical Pharmacogenetic Models of Treatment Response to Methotrexate Monotherapy in Slovenian and Serbian Rheumatoid Arthritis Patients: Differences in Patient's Management May Preclude Generalization of the Models

**DOI:** 10.3389/fphar.2018.00020

**Published:** 2018-01-25

**Authors:** Barbara Jenko, Matija Tomšič, Biljana Jekić, Vera Milić, Vita Dolžan, Sonja Praprotnik

**Affiliations:** ^1^Pharmacogenetics Laboratory, Faculty of Medicine, Institute of Biochemistry, University of Ljubljana, Ljubljana, Slovenia; ^2^Department of Rheumatology, University Medical Centre Ljubljana, Ljubljana, Slovenia; ^3^Faculty of Medicine, University of Ljubljana, Ljubljana, Slovenia; ^4^Faculty of Medicine, Institute of Human Genetics, University of Belgrade, Belgrade, Serbia; ^5^Faculty of Medicine, Institute of Rheumatology, University of Belgrade, Belgrade, Serbia

**Keywords:** rheumatoid arthritis, pharmacogenetics, methotrexate, polymorphism, predictive model

## Abstract

**Objectives:** Methotrexate (MTX) is the first line treatment for rheumatoid arthritis (RA), but nevertheless 30% of patients experience MTX inefficacy. Our aim was to develop a clinical pharmacogenetic model to predict which RA patients will not respond to MTX monotherapy. We also assessed whether this model can be generalized to other populations by validating it on a group of Serbian RA patients.

**Methods:** In 110 RA Slovenian patients, data on clinical factors and 34 polymorphisms in MTX pathway were analyzed by Least Absolute Shrinkage and Selection Operator (LASSO) penalized regression to select variables associated with the disease activity as measured by Disease Activity Score (DAS28) score after 6 months of MTX monotherapy. A clinical pharmacogenetic index was constructed from penalized regression coefficients with absolute value above 0.05. This index was cross-validated and also independently validated on 133 Serbian RA patients.

**Results:** A clinical pharmacogenetic index for prediction of DAS28 after 6 months of MTX monotherapy in Slovenian RA patients consisted of DAS28 score at diagnosis, presence of erosions, MTX dose, Solute Carrier Family 19 Member 1 (*SLC19A1*) rs1051266, Solute Carrier Organic Anion Transporter Family Member 1B1 (*SLCO1B1*) rs2306283, Thymidylate Synthase (*TYMS*), and Adenosine Monophosphate Deaminase 1 (*AMPD1*) rs17602729. It correctly classified 69% of Slovenian patients as responders or nonresponders and explained 30% of variability in DAS28 after 6 months of MTX monotherapy. Testing for validity in another population showed that it classified correctly only 22.5% of Serbian RA patients.

**Conclusions:** We developed a clinical pharmacogenetic model for DAS28 after 6 months of MTX monotherapy in Slovenian RA patients by combining clinical and genetic variables. The clinical pharmacogenetic index developed for Slovenian patients did not perform well on Serbian patients, presumably due to the differences in patients' characteristics and clinical management between the two groups.

## Introduction

Personalized medicine strives to prescribe the right drug for the right patient at the time of diagnosis. Clinical pharmacogenetic models are aimed at predicting the treatment response at the time of diagnosis, thus translating pharmacogenomics research into clinical practice. As many clinical, demographic and genetic factors influence the treatment response in multifactorial diseases, such as rheumatoid arthritis (RA), both clinical and genetic factors need to be included into the clinical pharmacogenetic model to correctly predict the treatment response. RA is a systematic autoimmune disease which may cause progressive joint damage, cartilage, and bone destruction. The goal of the treatment is to achieve remission or low disease activity to prevent the occurrence of severe joint damage. The European League Against Rheumatism (EULAR) guidelines recommend MTX as the first line treatment for RA (Smolen et al., 2014). However, 30% of patients experience MTX inefficacy or toxicity and the therapy should be modified by adding another disease-modifying antirheumatic drug (DMARD) or replacing one with another one. To prevent joint damage and erosions there is a need to identify patients with a higher risk to experience treatment inefficacy. With this aim the clinical pharmacogenetic model for the prediction of non-response to MTX treatment was introduced (Wessels et al., 2007) and validated (Fransen et al., 2012) in Dutch patients with an early onset of RA. It included female gender, DAS at baseline, rheumatoid factor (RF), smoking and Methylenetetrahydrofolate Dehydrogenase 1 (*MTHFD1*), Adenosine Monophosphate Deaminase 1 (*AMPD1*), Inosine Triphosphatase (*ITPA*), and 5-Aminoimidazole-4-carboxamide ribonucleotide Formyltransferase/IMP Cyclohydrolase (*ATIC*) polymorphisms. Although it resulted in a good prediction of non-responders, it is not widely used in clinical practice outside the Netherlands (Wessels et al., 2007; Fransen et al., 2012).

Since this first clinical pharmacogenetic model was introduced for RA, novel associations with the MTX treatment response were observed for many additional genetic factors that were not included in this initial model. In a genome-wide association study on MTX treatment efficacy, none of the polymorphisms crossed the strict level of significance (Senapati et al., 2014). However, after analyzing only genetic factors involved in MTX pathway, a rare *TYMS* rs34489327 polymorphism showed strong influence on nonresponse to MTX monotherapy (Senapati et al., 2014). The importance of other polymorphisms in *TYMS* was also observed in some reports (Jekic et al., 2013; Lima et al., 2014a). Additionally, several pharmacogenetics studies of adenosine pathway (Hider et al., 2008; Grabar et al., 2010; Varani et al., 2011; Lima et al., 2014b) were carried out, which is crucial for MTX anti-inflammatory actions. Apart from the associations between polymorphisms in folate and adenosine pathway with the MTX treatment response, also solute carrier SLCO1B1 as well as ABC transporters were shown to influence the MTX treatment response (Lima et al., 2014c; Romao et al., 2014; Zhu et al., 2014).

The aim of this study was to build the clinical pharmacogenetic model for the prediction of MTX treatment response in Slovenian RA patients by evaluating the combined effect of clinical and genetic factors on disease activity assessed by DAS28 and on the treatment response after 6 months of MTX monotherapy. We used LASSO penalized regression to develop a predictive model for DAS28 after 6 months of MTX monotherapy based on clinical factors and 34 polymorphisms in genes coding for MTX transporters, enzymes in folate and adenosine pathway and adenosine receptors. We cross-validated the developed index to obtain the predictive ability in Slovenian RA patients. We also assessed whether this index can be generalized to other populations by validating it in a group of Serbian RA patients.

## Materials and methods

### Study population and outcomes

All Serbian (Arnett et al., 1988) and Slovenian (Aletaha et al., 2010) patients fulfilled the 1987 revised American College of Rheumatology criteria for RA and were retrospectively recruited. RA patients were considered eligible for inclusion if they were initially treated with MTX monotherapy for at least 6 months, were over 18 years old and were of Central Caucasian origin. Slovenian patients were treated at the Department of Rheumatology, University Medical Centre, Ljubljana, Slovenia, while Serbian patients were treated at the Institute of Rheumatology, School of Medicine, University of Belgrade, Serbia.

Data on treatment duration with MTX as well as on RF and anti-citrullinated protein antibody (ACPA) positivity, folate supplementation, and the presence of erosive changes in hands and feet observed on X-rays were collected from individual patient's files.

The study was approved by the Republic of Slovenia National Medical Ethics Committee for research in medicine and by the Ethics Committee of the Institute of Rheumatology, Belgrade, Serbia, and was carried out according to the Declaration of Helsinki. All patients gave their written informed consent to participate in the study.

### Selection of polymorphisms

Sixteen candidate genes were selected for our study on the basis of their putative involvement in the MTX transport, folate, and adenosine pathway reported in literature (Mikkelsen et al., 2011). Polymorphisms identified in the National Centre for Biotechnology Information (NCBI) (Sherry et al., 2001) were selected in candidate genes and pathways previously associated with the MTX treatment response (Bohanec Grabar et al., 2008, 2012; Hider et al., 2008; Sharma et al., 2009; Romao et al., 2013; Umicevic Mirkov and Coenen, 2013; Lima et al., 2014a,c,d; Senapati et al., 2014). Based on the HapMap database (2003), putatively functional polymorphisms that tag haplotype blocks were also included. The functionality and haplotype-tagging of polymorphisms was explored using web-based prediction tools (Barrett et al., 2005; Yuan et al., 2006; Xu and Taylor, 2009; Boyle et al., 2012).

### Genotyping

Genomic DNA was isolated from peripheral venous blood as previously described (Bohanec Grabar et al., 2008). Investigated genes were selected based on their role in the MTX transport (11) and MTX metabolism (Malik and Ranganathan, 2013; Zhu et al., 2014), while the polymorphisms were selected based on their known role or known association with the MTX treatment in RA listed in Pharmacogenomics Knowledgebase (Whirl-Carrillo et al., 2012). All patients from the Slovenian group were genotyped for polymorphisms in genes coding for transporters: *SLC19A1* rs1051266 (p.His27Arg); *SLCO1B1*: rs2306283 (p.Asn130Asp), rs4149056 (p.Val174Ala), rs11045879 (c.1865+4846T>C); *ABCB1:* rs1045642 (p.Ile1145=), rs2032582 (p.Ser893Ala/Thr), rs1128503 (p.Gly412=); *ABCC2:* rs2804402 (c.-1019A>G), rs717620 (c.-24C>T), rs2273697 (p.Val417Ile) and *ABCG2*: rs2231137 (p.Val12Met), rs2231142 (p.Gln141Lys), folate pathway: *MTHFR*: rs1801133 (p.Ala222Val), rs1801131 (p.Glu470Ala); *MTR* rs1805087 (p.Asp975Gly); *MTRR* rs1801394 (p.Ile49Met); *TYMS:* rs34743033 (28-bp tandem repeats), rs34489327 (c.^*^449delAinsTTAAAG), adenosine pathway: *ADA* rs73598374 (p.Asp8Asn); *AMPD1* rs17602729 (p.Gln45Ter); *ATIC* rs2372536 (p.Thr116Ser); *ITPA* rs1127354 (p.Pro32Thr); *MTHFD1* rs2236225 (p.Arg653Gln) and tag single nucleotide polymorphisms in adenosine receptors: *ADORA2:* rs2298383 (c.-275+1797C>T), rs2236624 (c.333-527T>C), rs5751876 (p.Tyr361=), rs35320474 (c.^*^453_^*^454insT), rs17004921 (n.863-2568G>A) and *ADORA3:* rs3394 (c.^*^441A>G), rs2298191 (c.-2288A>G), rs3393 (c.^*^423G>A), rs35511654 (p.Ile103Leu), rs2229155 (p.Ala154=), rs1544223 (c.-581G>A). All patients from the Serbian group were genotyped for the following polymorphisms included in the model: *SLC19A1* rs1051266 (p.His27Arg); *SLCO1B1* rs2306283 (p.Asn130Asp); *ABCB1* rs1045642 (p.Ile1145=); *ABCC2* rs717620 (c.-24C>T); *TYMS* rs34743033 (28-bp tandem repeats) and *AMPD1* rs17602729 (p.Gln45Ter). Many of these patients were previously genotyped for *TYMS* rs34743033 polymorphism (Jekic et al., 2013).

Genotyping was performed using a fluorescence based competitive allele-specific assay (KASPar, Kbiosciences, Hoddesdon, UK). Allelic discrimination was performed as recommended by the manufacturer using ABI 7500HT (Applied Biosystems, CA, USA). Respective positive (samples with known genotype) and negative controls (no DNA) were included in the genotyping for each batch of samples. Furthermore, to ensure genotyping reproducibility, 20% of the samples were genotyped in duplicate and all the genotyping results were concordant.

### Statistical analyses

Categorical variables were summarized with frequency and percentage, while numerical variables were summarized with median and interquartile range (IQR, 25–75th percentile). Chi-square test was used to assess deviation of genotype frequency distribution from those expected under Hardy-Weinberg equilibrium (HWE). The dominant genetic model was used in all analyses.

Univariate linear regression was used to estimate the influence of individual polymorphisms and clinical covariates (baseline DAS28, sex, age, RF, or ACPA positivity, presence of erosions, disease duration before beginning of MTX treatment, MTX dose, folate supplementation, MTX monotherapy) on DAS28 after 6 months. Results are reported as *p*-value, regression coefficient (B), and 95% confidence interval of B (95%CI). A significance level of α = 0.05 was used in univariate analyses.

For model building, LASSO penalized regression was used, where the amount of shrinkage was determined by a tuning parameter λ, which was estimated by cross-validating the (partial) likelihood (Goeman, 2010; Pavlou et al., 2015). By using LASSO penalized regression, we have avoided overfitting of the data and biased results due to co-linearity of the included factors. The variables whose penalized regression coefficients were not shrunk to zero were considered as associated with DAS28 after 6 months of MTX monotherapy. LASSO shrinkage causes the estimates of the non-zero coefficients to be biased toward zero, which results in worse in-sample performance (lower R-squared) with the aim to get more realistic out-of-sample behavior, and thus a better validity in Serbian population. The clinical pharmacogenetic predictive index was calculated by the linear combination of the estimated penalized regression coefficients. Only polymorphisms with absolute effect size of more than 0.05 were included in the final index. Polymorphisms with penalized regression coefficients lower than 0.05 were considered to have too low an effect on DAS28 to be clinically important and were not included in the index. The predictive ability and explained variability were assessed by Pearson's correlation between observed DAS28 and predicted DAS28. Five-fold cross-validation was used to estimate the explained variability of the index on Slovenian patients. The clinical pharmacogenetic index was independently validated on Serbian patients.

To assess the percentage of correctly classified patients, sensitivity, specificity, positive predictive value (PPV), and negative predictive value (NPV) for moderate or poor treatment response were evaluated using EULAR response criteria (Fransen and van Riel, 2009). The treatment response was defined as good, moderate or poor. Good responders had DAS28 below 3.2 after 6 months of treatment and improvement in DAS28 of more than 1.2 in the first 6 months of MTX treatment. Moderate or poor responders had DAS28 above 3.2 and a reduction of DAS28 of <1.2 at 6 months of MTX treatment. The percentage of correctly classified patients, PPV, NPV, specificity, and sensitivity were assessed by comparing the observed and the predicted treatment responses.

All statistical analyses were carried out using the R software and package “penalized” was used for LASSO penalized regression (Goeman, 2010; R Core Team, 2014).

## Results

### Patient characteristics

The study group consisted of 110 Slovenian RA patients, while the validation group consisted of 133 Serbian RA patients. Demographic and clinical characteristics of all patients are listed in Table 1. The median age, sex, and ACPA positivity were not significantly different between both groups. Slovenian patients were treated with median MTX dose of 20 (15–20) mg/week, while Serbian patients received median MTX dose of 12.5 (10–15) mg/week (*p* < 0.0001). All Slovenian patients received folate supplementation, as compared to only 73% of Serbian patients on folate supplementation (*p* < 0.0001). Erosions were present in 39% of Slovenian patients and in 69.2% of Serbian patients (*p* = 0.004). RF positivity was present in 69% of Slovenian patients and in 84.5% of Serbian patients (*p* < 0.0001). Median DAS28 at the beginning of MTX treatment was 5.2 (4.4–5.9) in Slovenian patients and thus significantly lower compared to Serbian patients with DAS28 7.5 (6.8–8.2) (*p* < 0.0001). DAS28 remained significantly lower in Slovenian patients also after 6 months of MTX treatment being 3 (2.4–4.6) as compared to 4.3 (3.6–5.7) in Serbian patients (*p* < 0.0001). After 6 months of MTX monotherapy, more Slovenian patients achieved a good treatment response, while most Serbian patients achieved only a moderate treatment response (*p* = 0.0018).

**Table 1:** Characteristics of rheumatoid arthritis patients.

	**Slovenian group *N* = 110**	**Serbian group *N* = 133**	***p*-value**
Female gender, n (%)	89 (81)	97 (72.9)	0.14
Age at the beginning of MTX treatment (years)	58 (45–69)	56 (50–64)	0.95
MTX treatment duration (months)	28.6 (12–59.3)	6 (6–24)	<0.0001
MTX dose (mg/week)	20 (15–20)	12.5 (10–15)	<0.0001
Erosions, n (%)	43 (39)	92 (69.2)	<0.0001
ACPA positivity, n (%)^a^	81 (73.6)	48 (70.6)	0.65
RF positivity, n (%)^b^	76 (69)	109 (84.5)	0.004
Folate supplementation, n (%)	110 (100)	97 (73)	<0.0001
DAS28 at beginning of treatment	5.2 (4.4–5.9)	7.5 (6.8–8.2)	<0.0001
DAS28 after 6 months of treatment	3 (2.4–4.6)	4.3 (3.6–5.7)	<0.0001
Treatment response after 6 months of MTX monotherapy, n (%)			
Poor	28 (25.4)	15 (11.3)	0.0018
Moderate	22 (20)	95 (71.4)	
Good	60 (54.6)	23 (17.3)	

*Data are presented as frequencies (percentages) for categorical variables and medians and interquartile range (IQR, 25th to 75th percentile) for numerical variables*.

*MTX: Methotrexate; RF: Rheumatoid factor, ACPA: Anti-citrullinated protein antibodies*.

a *Data are available for all Slovenian and 68 Serbian patients*.

b *Data are available for all Slovenian and 129 Serbian patients*.

All Slovenian patients were genotyped for 34 polymorphisms in folate and adenosine pathway and all genotype frequencies were in HWE (*p* > 0.05), except for *MTHFR* rs1801131, which was excluded from the analysis (Supplementary Table 1). Serbian patients were genotyped for six polymorphisms, four of them were included in the clinical pharmacogenetic index. All genotype frequencies were in HWE (p > 0.05), with the exception of *SLC19A1* rs1051266, but its allele frequencies were comparable to frequencies in Slovenian patients (Supplementary Table 2).

### Clinical pharmacogenetic model for disease activity as measured by DAS28 after 6 months of MTX monotherapy

In univariate analysis of Slovenian patients the following clinical factors showed a significant association with higher DAS28 after 6 months of MTX monotherapy: higher DAS28 at diagnosis (*p* = 0.0035; adjusted *R*^2^ = 0.068), higher occurrence of erosions (*p* = 0.031, adjusted *R*^2^ = 0.034) and higher MTX dose (*p* < 0.001; adjusted *R*^2^ = 0.13; Supplementary Table 1). Univariate analysis of genetic factors showed that carriers of polymorphic *SLC19A1* rs1051266 A allele (*p* = 0.024; adjusted *R*^2^ = 0.037) or *ABCC2* rs717620 A allele (*p* = 0.044 adjusted *R*^2^ = 0.037) experienced significantly lower DAS28 after 6 months of MTX monotherapy, while carriers of polymorphic *SLCO1B1* rs2306283 G allele (*p* = 0.03, adjusted *R*^2^ = 0.034) or *AMPD1* rs17602729 T allele (*p* = 0.047, adjusted *R*^2^ = 0.027) had significantly higher DAS28 after 6 months of MTX monotherapy (Supplementary Table 1).

A clinical pharmacogenetic model was constructed by LASSO penalized regression for Slovenian RA patients. Significant associations with DAS28 after 6 months of MTX monotherapy included DAS28 at diagnosis (*B* = 0.15), presence of erosions (*B* = 0.04), MTX dose (*B* = 0.12), *SLC19A1* rs1051266 (*B* = −0.2), *SLCO1B1* rs2306283 (*B* = 0.22), *ABCB1* rs1045642 (*B* = 0.04), *ABCC2* rs717620 (*B* = −0.03), *TYMS* rs347430033 (*B* = 0.08), and *AMPD1* rs17602729 (*B* = 0.21). Using the estimated regression coefficients of selected variables with absolute effect size above 0.05, we developed a clinical pharmacogenetic index. *ABCB1* rs1045642 and *ABCC2* rs717620 had estimated penalized regression coefficients lower than 0.05, and were thus considered to be too low to be clinically important. The definite clinical pharmacogenetic index consisted of DAS28 at diagnosis, presence of erosions, MTX dose, *SLC19A1* rs1051266, *SLCO1B1* rs2306283, *TYMS* rs347430033, and *AMPD1* rs17602729 as shown in Table 2. Correlation between observed and predicted DAS28 after 6 months of MTX monotherapy was highly significant (*p* < 0.0001, ρ = 0.55; 95% CI:0.40; 0.67), with 30.3% of variability explained. Fivefold cross-validation of this correlation showed a similar correlation (ρ = 0.54) and 29% of explained variability. For the prediction of moderate or poor treatment response in Slovenian RA patients, a clinical pharmacogenetic index correctly classified 69% of patients and showed 62% PPV, 80% NPV, 82% sensitivity, and 58% specificity (Table 3).

**Table 2:** Clinical pharmacogenetic index obtained from Lasso penalized regression analysis to predict DAS28 after six months of methotrexate monotherapy and the corresponding measures of variability obtained by univariate analysis in Slovenian and Serbian patients.

**Factor**		**Slovenian group**				**Serbian group**		
		**Coefficients for clinical pharmacogenetic index**	**Univariate analysis**			**Univariate analysis**		
			***p*-value**	**B (95%CI)**	**Adjusted R^2^**	***p*-value**	**B (95%CI)**	**Adjusted R^2^**
DAS28 at diagnosis		0.15	0.0035	0.036 (0.12; 0.60)	0.068	<0.0001	0.93 (0.66; 1.20)	0.26
Erosions		0.04	0.031	0.67 (0.06;1.29)	0.034	0.16	0.42 (−0.16; 1.01)	0.008
MTX dose		0.12	<0.001	0.21 (0.11; 0.30)	0.13	0.03	−0.10 (−0.18; −0.008)	0.027
*SLC19A1* rs1051266	GA/AA	−0.2	0.024	−0.72 (−0.34; −0.09)	0.037	0.91	0.04 (−0.66; 0.74)	−0.01
	GG	0						
*SLCO1B1* rs2306283	AG/GG	0.22	0.03	0.68 (0.07; 1.29)	0.034	0.24	−0.38 (−1.02; 0.25)	0.003
	AA	0						
*TYMS* rs347430033	2R3R/3R3R	0.08	0.089	0.58 (−0.09; 1.25)	0.017	0.74	−0.11 (−0.77; 0.54)	−0.006
	2R2R	0						
*AMPD1* rs17602729	CT/TT	0.21	0.047	0.70 (0.01; 1.38)	0.027	0.01	−0.75 (−1.32; −0.18)	0.04
	CC	0						
Constant	/	0.29	/	/	/			

Predicted DAS28 after 6 months of MTX monotherapy = 0.29+DAS28^*^0.15 + presence of erosions ^*^0.04 + MTX dose ^*^0.12 + SLC19A1 rs1051266^*^(−0.2) + SLCO1B1 rs2306283^*^0.22 + TYMS rs347430033^*^0.08 + AMPD1 rs17602729^*^0.21.

**Table 3:** Observed and predicted treatment response in Slovenian and Serbian RA patients.

**Observed**	**Predicted**	**Slovenian patients (*n* = 110)**	**Serbian patients (*n* = 133)**
**GOOD**			
	Good	35 (31.8 %)	21 (15.8%)
	Moderate or poor	25 (22.7%)	2 (1.5%)
**MODERATE OR POOR**			
	Good	9 (8.2%)	101 (75.9%)
	Moderate or poor	41 (37.3%)	9 (6.8%)

We assessed the applicability of our clinical pharmacogenetic index for an independent group of Serbian RA patients. In univariate analysis, higher DAS28 at diagnosis (*p* < 0.0001, adjusted *R*^2^ = 0.26) was associated with higher DAS28 after 6 months of MTX monotherapy, while a higher MTX dose (*p* = 0.03; adjusted *R*^2^ = 0.027) was associated with lower DAS28 after 6 months of MTX monotherapy (Supplementary Table 2). Among genetic factors, only *AMPD1* rs17602729 (*p* = 0.01; adjusted *R*^2^ = 0.04) was associated with DAS28 after 6 months of MTX monotherapy. The correlation between observed DAS28 and predicted DAS28 was not significant in the Serbian patient group (*p* = 0.53; ρ = −0.054; 95% CI −0.22; 0.12). The prediction of moderate or poor treatment response correctly classified 22.5% of patients and showed 82% PPV, 17% NPV, 8% sensitivity, and 91% specificity (Table 3).

## Discussion

We developed clinical pharmacogenetic index for the prediction of DAS28 after 6 months of MTX monotherapy in Slovenian RA patients consisting of DAS28 at diagnosis, presence of erosions, MTX dose, *SLC19A1* rs1051266, *SLCO1B1* rs2306283, *TYMS* rs347430033, and *AMPD1* rs17602729. This index correctly classified 69% of Slovenian RA patients and explained 30% of variability in DAS28 after 6 months of MTX monotherapy. After cross-validation, clinical pharmacogenetic index still explained 29% of predicted variability in DAS28 after 6 months of MTX monotherapy. In this model, the greatest part of variability was explained by MTX dose (13%) and DAS28 at diagnosis (6.8%). However, when we tested if the developed index can be generalized to other patient groups, we observed that this clinical pharmacogenetic index correctly classified only 22.5% of Serbian RA patients and thus poorly predicted DAS28 after 6 months of MTX monotherapy in another population.

Among genetic factors, *SLC19A1* rs1051266 A allele was associated with lower DAS28 after 6 months of MTX monotherapy. *SLC19A1* codes for reduced folate carrier which imports MTX into the cell, so higher MTX influx caused by *SLC19A1* rs1051266 A allele (Baslund et al., 2008) led to lower DAS28 after 6 months of MTX monotherapy. The studies investigating the influence of this polymorphism on MTX treatment outcome are not concordant (Lima et al., 2014c), but meta-analysis confirmed the influence of AA genotype on MTX efficacy in recessive and additive model (Kung et al., 2014). *SLCO1B1* rs2306283was associated with higher DAS28 after 6 months of MTX monotherapy in Slovenian, but not Serbian RA patients. Also, the only previously published study observed no effect of this polymorphism on MTX efficacy in RA (Lima et al., 2014c). Nevertheless, our observation warrants further investigation as *SLCO1B1* rs2306283 GG genotype was associated with higher transport activity and lower plasma MTX concentration in children treated for acute lymphoblastic leukemia (Ramsey et al., 2012; Giacomini et al., 2013). In Slovenian RA patients, we also observed higher DAS28 after 6 months of MTX monotherapy among homozygous carriers of three tandem repeats of 28 base pairs (3R) in *TYMS* gene (rs347430033) as compared to homozygous or heterozygotes carriers of two tandem repeats (2R). *TYMS* 3R/3R genotype leads to higher thymidylate synthase levels and was already associated with higher disease activity and non-response to MTX treatment in RA (Dervieux et al., 2004; Lima et al., 2014a). Carriers of *AMPD1* rs17602729 T allele also experienced higher DAS28 after 6 months of MTX monotherapy. This association had a strong effect in the clinical pharmacogenetic model, but was marginal in univariate analysis in Slovenian patients. On the contrary, univariate analysis in Serbian patients showed significant influence of *AMPD1* rs17602729 T allele on lower DAS28 after 6 months of MTX monotherapy. This association was also observed and included in the Dutch clinical pharmacogenetic model (Wessels et al., 2006), however, it was not supported by other studies (Fransen et al., 2012; Owen et al., 2013). It seems that the association between *AMPD1* rs17602729 and MTX treatment efficacy is influenced by confounding variables such as MTX dose or DAS28 at diagnosis.

The developed model showed a good prediction of DAS28 after 6 months of MTX monotherapy for Slovenian patients, also after cross-validation, but the model poorly predicted DAS28 after 6 months of MTX monotherapy for Serbian patients. Our model included all factors, except for smoking, that were included into previously published Dutch clinical pharmacogenetic model (Wessels et al., 2007). Due to the lack of data on smoking in our study group, we could not evaluate the Dutch model by using our data. By including more variables in the analysis and by using penalized regression, we managed to correctly classify 69% of Slovenian patients, which is more than in the Dutch model (52.7% in testing and 49% in validation group; Wessels et al., 2007; Fransen et al., 2012) and comparable to predictive model of clinical non-response to MTX treatment in juvenile idiopathic arthritis (72% in testing and 65% in validation group; Bulatovic et al., 2012). However, the index developed for Slovenian patients performed poorly in Serbian patients as it correctly classified only 22.5% of these patients. This could be due to significant differences in the clinical management between both study groups. Serbian patients could have significantly more active disease and higher DAS28 at diagnosis than Slovenian patients because they were managed according to 1987 revised American College of Rheumatology criteria for RA (Arnett et al., 1988). On the other hand, Slovenian patients were diagnosed earlier (Aletaha et al., 2010) and treated more aggressively as suggested by the recent EULAR guidelines (Smolen et al., 2014). This is also reflected in MTX doses, with higher MTX doses reaching the median of 20 mg/week in Slovenian patients, while Serbian patients were treated with lower MTX doses up to 15 mg/week.

If we compare our clinical pharmacogenetic index with the one developed and validated in the Dutch RA patients, we can see that disease activity as measured by DAS28 at the beginning of treatment and *AMPD1* polymorphism were included in both models; however, the effect of *AMPD1* rs17602729 differed between both groups. It seems that the disease activity as measured by DAS28 at the beginning of MTX treatment is a uniform predictor of MTX treatment efficacy among all patients groups, while other factors may vary among populations due to confounding variables related to treatment management. By adjusting R^2^, DAS28 at diagnosis with MTX dose explained the highest proportion of variability. In the prediction index for Slovenian patients, genetic factors explain a small but non-negligible proportion of variability, so we assume that the proposed predictive index might be cost-effective. The importance of both pharmacogenetic and clinical factors has been already shown in leukemia treatment, with *SLCO1B1* genetic variability explaining 10.7% and treatment arm explaining ~17% of interpatient variability in MTX clearance among patients with acute lymphoblastic leukemia (Ramsey et al., 2012). Considering our results and the results of the Dutch studies (Bulatovic et al., 2012; Fransen et al., 2012), we may conclude that valuable clinical pharmacogenetic index for the prediction of MTX efficacy should be developed and validated on a genetically homogenous patients group undergoing similar treatment management.

Our study had some limitations and there is some room for improvement in the prediction ability of clinical pharmacogenetic index. Retrospective data collection could have introduced bias. Data on smoking that would be valuable information for validating previously published clinical pharmacogenetic index and improving the predictive ability of our clinical pharmacogenetic index were not available. By including only polymorphisms in MTX transport and MTX metabolic pathways we may have missed other polymorphisms that may influence MTX treatment response (Aslibekyan et al., 2014) as well as rare mutations which may influence MTX disposition in a non-negligible percentage of patients (Ramsey et al., 2012). Also, the sample size was rather small. However, within the inclusion period, we involved all patients that fulfilled the inclusion criteria, had all data available and were willing to participate, so the number of participants was not based on power calculations. Further improvements could also be made by the investigation of SNPs influence on MTX pharmacokinetics; however, we did not have data on MTX plasma levels. The main strength of our study is the comprehensive approach to the variable selection and advanced statistical analysis, by which we tried to avoid model overfitting due to many variables in a moderate data set. For Slovenian as well as for Serbian patients, the analyses were not biased by genetic heterogeneity since the patients of both groups were recruited in geographical areas with ethnically homogeneous population. Furthermore, the treatment and follow-up were centralized for each group of RA patients.

In conclusion, we successfully built the first clinical pharmacogenetic index of MTX treatment response in Slovenian RA patients that directly predicts DAS28 after 6 months of MTX monotherapy by combining clinical and genetic variables. We showed that pharmacogenetic factors have small but non-negligible predictive value. The described approach using penalized regression may serve as an example how to select important variables with true effect and how to avoid model overfitting. Cross-validation showed good potential for prediction of DAS28 after 6 months of MTX monotherapy in Slovenian patients. We assessed if this clinical pharmacogenetic index can be applied in Serbian RA patients. However, the clinical pharmacogenetic index developed for Slovenian patients did not perform well on Serbian patients, presumably due to the differences in patients' characteristics and clinical management between the two groups. Since the generalization of the presented clinical pharmacogenetic index is sensitive to treatment management, population-based clinical pharmacogenetic indices should be developed for populations with different treatment approaches.

## Author contributions

BaJ, VD, MT, and SP: Conception and design; BaJ and VD: Development of methodology; BaJ, VD, SP, BiJ, and VM: Acquisition of data; BaJ and VD: Analysis and interpretation of data; BaJ, VD, SP, MT, BiJ, and VM: Drafting and writing of the manuscript.

### Conflict of interest statement

The authors declare that the research was conducted in the absence of any commercial or financial relationships that could be construed as a potential conflict of interest.
