# Supplementary material for: Clinical Pharmacogenetic Models of Treatment Response to Methotrexate Monotherapy in Slovenian and Serbian Rheumatoid Arthritis Patients: Differences in Patient's Management May Preclude Generalization of the Models
Source: Front Pharmacol. 2018 Jan 25;9:20. doi: 10.3389/fphar.2018.00020 (PMC5788961; doi:10.3389/fphar.2018.00020)
Supplement: Supplementary file 1 [file Table1.docx]

Supplementary table 1: Association of clinical variables and polymorphisms with DAS28 after 6 months of MTX monotherapy. Results are obtained from univariate cause-specific Cox models, and LASSO penalized regression in the cause-specific Cox proportional hazard model with polymorphisms and clinical factors included.

|  | | | | | Univariate analysis | | Lasso penalized regression |
| --- | --- | --- | --- | --- | --- | --- | --- |
|  |  |  |  |  | B (95%CI) | p-value | B |
| DAS28 at diagnosis | | | | | 0.36 (0.12; 0.6) | 0.0035 | 0.16 |
| Female gender | | | | | 0.04 (-0.73; 0.81) | 0.91 | 0 |
| Age | | | | | 0.016 (-0.005; 0.04) | 0.14 | 0 |
| Disease duration before MTX treatment | | | | | 0.005 (-0.23; 0.03) | 0.71 | 0 |
| Erosions | | | | | 0.67 (0.06; 1.29) | 0.03 | 0.04 |
| RF positivity | | | | | 0.25 (-0.40; 0.91) | 0.44 | 0 |
| ACPA positivity | | | | | 0.06 (-0.63; 0.75) | 0.85 | 0 |
| MTX dose | | | | | 0.21 (0.11; 0.30) | 5.83 e^-5^ | 0.12 |
| Factor | genotype | n | HW | MAF |  |  |  |
| *SLC19A1*  rs1051266 | GG | 39 | 0.64 | 0.18 | -0.72 (-1.34; -0.09) | 0.024 | -0.19 |
|  | GA/AA | 71 |  |  |  |  |  |
| *ABCB1*  rs1128503 | CC | 32 | 0.67 | 0.18 | 0.29 (-0.37; 0.96) | 0.38 | 0 |
|  | CT/TT | 74 |  |  |  |  |  |
| *ABCB1*  rs2032582 | GG | 36 | 0.63 | 0.16 | 0.07 (-0.58; 0.72) | 0.84 | 0 |
|  | GT/ GA/TT/AA | 86 |  |  |  |  |  |
| *ABCB1*  rs1045642 | CC | 24 | 0.25 | 0.22 | 0.44 (-0.29; 1.18) | 0.23 | 1.04 |
|  | CT/TT | 86 |  |  |  |  |  |
| *SLCO1B1*  rs4149056 | TT | 70 | 0.18 | 0.02 | 0.13 (-0.5; 0.76) | 0.67 | 0 |
|  | TC/CC | 40 |  |  |  |  |  |
| *SLCO1B1*  rs11045879 | TT | 69 | 0.35 | 0.03 | 0.04 (-0.58; 0.67) | 0.88 | 0 |
|  | TC/CC | 41 |  |  |  |  |  |
| *SLCO1B1*  rs2306283 | AA | 42 | 0.29 | 0.11 | 0.68 (0.06; 1.29) | 0.03 | 1.25 |
|  | AG/GG | 68 |  |  |  |  |  |
| ABCG2  rs2231137 | GG | 97 | 0.06 | 0.12 | 0.04 (-0.90; 0.98) | 0.93 | 0 |
|  | GA | 13 |  |  |  |  |  |
| ABCG2  rs2231142 | CC | 94 | 0.64 | 0.009 | -0.46 (-1.32; 0.39) | 0.29 | 0 |
|  | CA/AA | 16 |  |  |  |  |  |
| *ABCC2*  rs2804402 | CC | 19 | 0.01 | 0.17 | 0.02 (-0.78;0.82) | 0.96 | 0 |
|  | CT/TT | 91 |  |  |  |  |  |
| *ABCC2*  rs717620 | GG | 67 | 0.56 | 0.04 | -0.63 (-1.23;-0.02) | 0.04 | -0.03 |
|  | GA/AA | 43 |  |  |  |  |  |
| *ABCC2*  rs2273697 | GG | 56 | 0.99 | 0.08 | 0.03 (-0.58; 0.65) | 0.92 | 0 |
|  | GA/AA | 54 |  |  |  |  |  |
| *ADORA2A*  rs2298383 | TT | 59 | 0.28 | 0.1 | -0.10  (-0.71; 0.51) | 0.75 | 0 |
|  | TC/CC | 51 |  |  |  |  |  |
| *ADORA2A*  rs2236624 | CC | 70 | 0.47 | 0.03 | -0.06 (-0.69; 0.57) | 0.84 | 0 |
|  | CT/TT | 40 |  |  |  |  |  |
| *ADORA2A*  rs5751876 | CC | 53 | 0.27 | 0.13 | -0.03 (-0.63; 0.57) | 0.91 | 0 |
|  | CT/TT | 57 |  |  |  |  |  |
| *ADORA2A*  rs35320474 | TT | 53 | 0.27 | 0.13 | -0.04 (-0.64; 0.57) | 0.91 | 0 |
|  | T+del/ del+del | 57 |  |  |  |  |  |
| *ADORA2A*  rs17004921 | CC | 87 | 0.76 | 0.009 | 0.37 (-0.37; 1.12) | 0.33 | 0 |
|  | CT/TT | 23 |  |  |  |  |  |
| *ADORA3*  rs3394 | CC | 66 | 0.48 | 0.04 | 0.04 (-0.57; 0.67) | 0.88 | 0 |
|  | CT/TT | 44 |  |  |  |  |  |
| *ADORA3*  rs2298191 | TT | 56 | 0.99 | 0.08 | 0.38 (-0.23; 0.98) | 0.22 | 0 |
|  | CT/CC | 54 |  |  |  |  |  |
| *ADORA3*  rs3393 | AA | 37 | 0.89 | 0.18 | 0.01 (-0.63; 0.65) | 0.97 | 0 |
|  | AG/GG | 73 |  |  |  |  |  |
| *ADORA3*  rs35511654 | TT | 82 | 0.97 | 0.02 | 0.14 (-0.55; 0.84) | 0.68 | 0 |
|  | TG/GG | 28 |  |  |  |  |  |
| *ADORA3*  rs2229155 | CC | 66 | 0.49 | 0.04 | 0.04 (-0.57; 0.66) | 0.88 | 0 |
|  | CT/TT | 44 |  |  |  |  |  |
| *ADORA3*  rs1544223 | AA | 57 | 0.19 | 0.05 | 0.24 (-0.36; 0.85) | 0.43 | 0 |
|  | AG/GG | 53 |  |  |  |  |  |
| *MTHFR*  rs1801133 | CC | 53 | 0.82 | 0.1 | 0.48 (-0.12; 1.08) | 0.12 | 0 |
|  | CT/TT | 57 |  |  |  |  |  |
| *MTHFR*  rs1801131 | AA | 54 | 0.005 | 0.02 | -010 (-0.71; 0.50) | 0.73 | 0 |
|  | AC/CC | 56 |  |  |  |  |  |
| *MS*  rs1805087 | AA | 66 | 0.25 | 0.03 | -0.07 (-0.68; 0.55) | 0.83 | 0 |
|  | AG/GG | 44 |  |  |  |  |  |
| *MTRR*  rs1801394 | AA | 29 | 0.25 | 0.3 | -0.23 (-0.99; 0.39) | 0.39 | 0 |
|  | AG/GG | 81 |  |  |  |  |  |
| *TYMS* rs34743033 | 2R+2R | 80 | 0.44 | 0.26 | 0.57 (-0.09; 1.25) | 0.08 | 1.08 |
|  | 2R+3R/3R+3R | 30 |  |  |  |  |  |
| *TYMS*  rs34489327 | del | 108 | 0.92 | 0.02 | -0.10 (-2.38; 2.18) | 0.93 | 0 |
|  | del/TTAAAG | 2 |  |  |  |  |  |
| *ADA*  rs73598374 | GG | 100 | 0.62 | 0 | 0.61 (-0.40; 1.62) | 0.23 | 0 |
|  | GA/AA | 10 |  |  |  |  |  |
| *AMPD1*  rs17602729 | CC | 82 | 0.52 | 0.03 | 0.69 (0.01; 1.38) | 0.04 | 0.21 |
|  | CT/TT | 28 |  |  |  |  |  |
| *ATIC*  rs2372536 | CC | 45 | 0.24 | 0.09 | -0.15 (-0.77; 0.47) | 0.63 | 0 |
|  | CG/GG | 65 |  |  |  |  |  |
| *ITPA*  rs1127354 | CC | 94 | 0.41 | 0 | -0.25 (-1.12; 0.61) | 0.56 | 0 |
|  | CA/AA | 16 |  |  |  |  |  |
| *MTHFD1*  rs2236225 | GG | 41 | 0.76 | 0.16 | -0.28 (-0.92; 0.35) | 0.38 | 0 |
|  | GA/AA | 69 |  |  |  |  |  |

HW: Hardy–Weinberg equilibrium, MAF: Minor allele frequency

Supplementary table 2: Genotype frequencies for Serbian patients and results of univariate analysis association between various factors with DAS28 after six months of MTX monotherapy.

| **Factor** | **Genotype** | **n** | **HW** | **MAF** | **p-value** | **B (95%CI)** |
| --- | --- | --- | --- | --- | --- | --- |
| DAS28 at diagnosis | | | | | <0.0001 | 0.93 (0.66; 1.20) |
| Erosions | | | | | 0.16 | 0.42 (-0.16; 1.01) |
| MTX dose | | | | | 0.03 | -0.10 (-0.18; -0.008) |
| *SLC19A1* rs1051266 | GG  GA/AA | 25  108 | 0.02 | 0.21 | 0.91 | 0.04 (-0.66; 0.74) |
| *ABCB1* rs1045642 | CC  CT/TT | 28  105 | 0.19 | 0.23 | 0.64 | 0.16 (-0.51; 0.83) |
| *SLCO1B1* rs2306283 | AA  AG/GG | 31  101 | 0.19 | 0.20 | 0.24 | -0.38 (-1.02; 0.25) |
| *ABCC2* rs717620 | GG  GA/AA | 90  43 | 0.26 | 0.06 | 0.54 | -0.16 (-0.71; 0.38) |
| *TYMS* rs347430033 | 2R2R  2R3R/3R3R | 30  103 | 0.97 | 0.27 | 0.74 | -0.11 (-0.77; 0.54) |
| *AMPD1* rs17602729 | CC  CT/TT | 91  42 | 0.99 | 0.03 | 0.01 | -0.75 (-1.32; -0.18) |

HW: Hardy–Weinberg equilibrium, MAF: Minor allele frequency
